# Assessing Repetitive Negative Thinking Using Categorical and Transdiagnostic Approaches: A Comparison and Validation of Three Polish Language Adaptations of Self-Report Questionnaires

**DOI:** 10.3389/fpsyg.2016.00322

**Published:** 2016-03-09

**Authors:** Monika Kornacka, Jacek Buczny, Rebekah L. Layton

**Affiliations:** ^1^PSITEC Lab, University of LilleLille, France; ^2^SWPS University of Social Sciences and HumanitiesSopot, Poland; ^3^University of North Carolina at Chapel HillChapel Hill, NC, USA

**Keywords:** perseverative thinking, rumination, worry, transdiagnostic process, mood disorder

## Abstract

Repetitive negative thinking (RNT) is a transdiagnostic process involved in the risk, maintenance, and relapse of serious conditions including mood disorders, anxiety, eating disorders, and addictions. Processing mode theory provides a theoretical model to assess, research, and treat RNT using a transdiagnostic approach. Clinical researchers also often employ categorical approaches to RNT, including a focus on depressive rumination or worry, for similar purposes. Three widely used self-report questionnaires have been developed to assess these related constructs: the Ruminative Response Scale (RRS), the Perseverative Thinking Questionnaire (PTQ), and the Mini-Cambridge Exeter Repetitive Thought Scale (Mini-CERTS). Yet these scales have not previously been used in conjunction, despite useful theoretical distinctions only available in Mini-CERTS. The present validation of the methods in a Polish speaking population provides psychometric parameters estimates that contribute to current efforts to increase reliable replication of theoretical outcomes. Moreover, the following study aims to present particular characteristics and a comparison of the three methods. Although there has been some exploration of a categorical approach, the comparison of transdiagnostic methods is still lacking. These methods are particularly relevant for developing and evaluating theoretically based interventions like concreteness training, an emerging field of increasing interest, which can be used to address the maladaptive processing mode in RNT that can lead to depression and other disorders. Furthermore, the translation of these measures enables the examination of possible cross-cultural structural differences that may lead to important theoretical progress in the measurement and classification of RNT. The results support the theoretical hypothesis. As expected, the dimensions of brooding, general repetitive negative thinking, as well as abstract analytical thinking, can all be classified as unconstructive repetitive thinking. The particular characteristics of each scale and potential practical applications in clinical and research are discussed.

## Introduction

Since the early nineties, the number of research papers on repetitive negative thinking (RNT) in clinical psychology has constantly grown. Today, RNT is known to be a key transdiagnostic process (i.e., a process involved in risk, maintenance, and reoccurrence of various psychological disorders; Ehring and Watkins, 2008; Nolen-Hoeksema and Watkins, 2011; Watkins, 2011). Development of rumination-focused therapy and initial empirical results suggesting its effectiveness provide evidence confirming the important role of RNT in clinical psychology (Watkins et al., 2009, 2011). Unfortunately, international instruments for the assessment of RNT such as those in Poland are very poorly developed or nonexistent, compared with the other countries conducting experimental and clinical research on this process despite this growing interest in both the Polish and international communities^1^. The validation of instruments diagnosing RNT could initiate a line of research examining possible structural differences in scales that might reflect theoretical and methodological innovations (Roger et al., 2001).

The emphasis put on the transdiagnostic approach in the current clinical and experimental research created the need for the transdiagnostic evaluation of the RNT. Consequently, several transdiagnostic methods were developed almost simultaneously (e.g., Mini-CERTS, PTQ). Those methods were often compared to the already existing categorical measures of RNT but very rarely to each other. Additionally, each of the transdiagnostic methods presents very particular characteristics that can be advantageous in both clinical and research settings. The present article compares three scales, including a classic tool to evaluate depressive rumination (RRS) and two newly developed transdiagnostic tools. To our knowledge, this is the first research linking two transdiagnostic scales.

RNT can be classified as an emotional regulation strategy involving self-focused attention, and characterized by repetitive, prolonged, and recurrent thoughts about one's experience and concerns (Lyubomirsky and Nolen-Hoeksema, 1995; Watkins, 2008). The research on RNT began with response style theory developed by Nolen-Hoeksema (1991). According to this theory rumination refers to “behaviors and thoughts that focus one's attention on one's depressive symptoms and on the implication of these symptoms” (Nolen-Hoeksema, 1991, p. 569). Response style theory postulates that rumination is an automatic response to activation of a dysphoric (depressive) mode, which over the long-term captures individuals in a vicious cycle where dysphoric mode triggers rumination, and rumination increases negative mood. Further studies on depressive rumination have suggested a distinction between brooding rumination—the passive comparison of one's current situation with unachieved standards, and reflection rumination—the purposeful focus on cognitive problem solving in order to alleviate one's depressive symptoms (Treynor et al., 2003). The literature clearly shows that brooding is a risk factor leading to maintenance and relapse of various psychological disorders like depression, anxiety, eating disorders, and addictions (Nolen-Hoeksema et al., 2008; Watkins, 2008). Recent studies suggest that brooding can also play the role of mediator between other risk factors and clinical disorders (O'Connor et al., 2007; Olson and Kwon, 2007; Macedo et al., 2015). There is no consistency in the research results concerning adaptive proprieties of reflection. Reflection correlates with brooding, but not always with psychological disorders, and some researchers postulate that reflection might in fact be an adaptive dimension of depressive rumination (Treynor et al., 2003).

Response style theory includes a wide body of research, however, taking that into account that by its very definition, depressive rumination is focused on depressive symptoms. Thus, the application of response style theory more widely to psychological disorders other than depression remains problematic. Addressing this issue, a new approach was proposed by Watkins and Teasdale (2004). According to processing mode theory the adaptive features of RNT do not depend on the content of thoughts (i.e., information itself, as in depressive rumination), but rather depend on the way information is processed (Watkins and Teasdale, 2004; Watkins, 2009). Processing mode theory states that in RNT information can be processed via two alternative modes, the *abstract analytic* or *concrete experiential* modes.

The *abstract analytic* (AA) mode implies analyzing the causes, consequences, and significance of an event or information, whereas the *concrete experiential* (CE) mode implies concrete, process-focused, and specific thinking about one's current emotional state, physical sensations, and the details of the environment (Watkins, 2009). One of the main differences that follows from this distinction between AA and CE processing modes is their respective temporal orientations. While AA thinking is focused on the past or on the future, CE thinking refers to the here-and-now. Nevertheless, both processing modes can be applied to the same event/situation. In agreement with action identification theory (Vallacher and Wegner, 1989), processing mode theory suggests that in a situation characterized by negative mood or unresolved problems, individuals switch to concrete processing to adaptively resolve the current situation. When the activated processing mode is no longer consistent with the ongoing situation, an RNT malfunction can occur (Watkins, 2011). Thus, maladaptive RNT will be caused primarily by the default use of abstract processing when it is not appropriate.

The main advantage of processing mode theory is that it is transdiagnostic in nature. The functional proprieties of RNT depend on the processing mode and its adjustment to the ongoing situation, rather than on the content of thoughts, *per se*. Consequently, the same process can be dysfunctional in a whole range of psychological disorders, regardless of the specific content of thoughts (e.g., dwelling on the past in depression, worries about the future in anxiety, or craving in addictions). According to previous research, the lack of flexibility in the use of the two processing modes, especially the automatic use of AA thinking as a coping strategy to alleviate negative emotions, can lead to emotional regulation impairment, reduce problem solving, and enhance cognitive biases (Watkins and Teasdale, 2001; Moberly and Watkins, 2006). Greater dependence on the AA processing mode is also correlated with report of more depressive symptoms (Watkins, 2004; Watkins et al., 2011).

Evidence suggests that RNT appears to be one of the most relevant processes in the transdiagnostic approach to treating a variety of disorders in clinical psychology (Ehring and Watkins, 2008; Nolen-Hoeksema and Watkins, 2011). Recently, a new cognitive–behavioral intervention (concreteness training) was developed and tested with very promising results, an intervention with a focus on RNT which is a key element of rumination-focused cognitive behavioral therapy. The aim of concreteness training is to enhance the flexible use of RNT processing modes, and to reinforce the use of the more adaptive CE mode (Watkins et al., 2009, 2011). Initial results suggest that concreteness training reduces depressive symptoms, overgeneralization of cognitive biases, and rumination (Watkins et al., 2011).

The increasing body of research on RNT in clinical psychology, in line with the development and evaluation of the clinical intervention focused on RNT, accordingly requires the use of similarly process-focused assessments. Development of RNT theory has thus naturally evolved to include the creation of self-report questionnaires for use in assessment of RNT theoretical models. The first scale to be developed assessing RNT was the Ruminative Response Scale evaluating depressive rumination (RRS; Nolen-Hoeksema, 1991). The main critique of this scale concerned the overlap between depressive rumination and depressive symptoms. To address this issue, the RRS was revised such that the items referring to depressive symptomatology, rather than referring directly to depressive rumination, were identified and removed. Principal components analysis in the revised version of the RRS revealed two dimensions of depressive rumination: brooding and reflection, corroborating ruminative response theory (Nolen-Hoeksema, 1991; Treynor et al., 2003).

Although the Ruminative Response Scale-Revised (RRS-R; Treynor et al., 2003) remains an excellent questionnaire to evaluate depressive rumination, its use in the context of other psychological disorders might be problematic. It seems likely that patients suffering from anxiety, addictions, or eating disorders might be focused on other preoccupations than their depressive symptoms. Among the scales assessing RNT, the RRS-R is the most used in research; it is however necessary to note that this scale was the first, and for years the only scale available to address evaluation of RNT from a clinical perspective, which might explain its use in various psychological disorders, regardless of depressive rumination definitions.

Subsequently, a scale assessing RNT within the transdiagnostic approach was developed, the Perseverative Thinking Questionnaire (PTQ) by Ehring et al. (2011). The PTQ is based on the assumption that rumination and worries, regardless of different temporal orientation, are the same process and can be classified and measured as one general process: repetitive negative thinking. Both rumination and worries are self-focused and abstract thoughts (Ehring and Watkins, 2008). They can be distinguished by their content and their temporal orientation—anticipation of the future for worries involved in anxiety, and dwelling in the past for rumination involved in depression, but the process of repetitive thinking itself remains transdiagnostic. Of note, this conceptualization is similar to processing mode theory in which unconstructive AA modes can be focused on the past, or on the future. Consequently, to assess a general use of RNT independently of its future/past temporal orientation or the content of specific psychological disorder, the items of the PTQ were created with both processes in mind. Therefore, the PTQ can be used to assess repetitive negative thinking including rumination (as in the RRS-R; Treynor et al., 2003) and worries (as in the Penn State Worry Questionnaire; Meyer et al., 1990). The fact that mood disorders and anxiety often appear as comorbid disorders is at least one reason for encouraging the search for one transdiagnostic process involved in both pathologies. As an added value, this combination approach makes the use of the PTQ of interest as a timesaving method in clinical practice. This is because the PTQ not only measures the general RNT, but can be also divided into three sub-dimensions characteristic of RNT from the clinical point of view: core features of RNT, unproductiveness of RNT, and mental capacity captured by RNT.

Another transdiagnostic measure of RNT has been developed recently as well. Similar to the PTQ, the Mini-Cambridge Exeter Repetitive Thought Scale (Mini-CERTS; Douilliez et al., 2014) assesses the RNT from a content and disorder independent perspective, but unlike the PTQ, its creation is not based on the cross section between worries and rumination, but on the processing mode theory itself. The Mini-CERTS was created by extracting the items evaluating abstract analytic and concrete experiential thinking from Cambridge Exeter Repetitive Thought Scale (Barnard et al., 2007). In accordance with the processing mode theory (Watkins and Teasdale, 2004) and reduced concreteness theory (Stober and Borkovec, 2002), the Mini-CERTS assesses the unconstructive (abstract analytic) repetitive thinking separately from the constructive (concrete experiential) repetitive thinking. An incontestable advantage of the Mini-CERTS is the assessment of both the adaptive CE and maladaptive AA forms of processing mode in RNT; this evaluation seems particularly interesting from the perspective of the RNT-based clinical intervention, concreteness training (Watkins et al., 2009, 2011).

The first aim of the present study was to compare the structures of two newly developed transdiagnostic scales evaluating the repetitive negative thinking in a transdiagnostic approach (Mini-CERTS and PTQ) with a self-reported method classically used to evaluate depressive rumination (RRS-R). The second aim was to evaluate the linguistic accuracy of the translation of the three scales presented above, to assess their psychometric properties in the Polish-language version, and to pave the way for future investigations of theoretical distinctions that may arise from multilingual comparisons.

The first step of the study was assessing the accuracy of the English-Polish translation and the correlation between the Polish-language and English-language versions of the questionnaires. In the second step, the back-translation of the questionnaire was compared to the original version. The internal consistency and concurrent validity of each scale was evaluated in the third step. Finally, the concurrent validity was evaluated by the correlations between dimensions of the three scales presented above. Although all three scales measure different aspects of RNT, the literature as well as RNT theory clearly suggest a positive correlation between each of the following pairs: brooding and abstract analytic thinking; brooding and repetitive negative thinking from the PTQ; abstract analytic thinking and repetitive negative thinking from the PTQ; and finally, a negative correlation between CE, the constructive processing mode of RNT, and abstract analytic thinking the unconstructive mode of RNT.

## Methods

### Participants

#### Sample 1

Twelve psychology students from the SWPS University of Social Sciences and Humanities (Sopot, Poland) each assessed the translation accuracy for one of three scales (4 participants for the RRS-R, 4 participants for the Mini-CERTS, and 4 participants for the PTQ). Students had each, at minimum, passed English language standardized tests in order to obtain college admission, successfully completed at least 2-years of required coursework in English to obtain senior standing, and were required to self-evaluate their proficiency in English as fluent in order to participate.

#### Sample 2

Two English-language experts who are native English speakers (one with a doctorate degree in psychology and one who is an editor with a master's degree in English literature) evaluated the back translation of the PTQ, the Mini-CERTS, and the RRS-R.

#### Sample 3

Participants (*n* = 107; 10 male, 97 female) filled in only the Polish-language (*n* = 60), or both Polish-language and English-language versions (*n* = 106, for the participants who self-evaluated their proficiency in English as fluent and fulfilled the same prerequisites mentioned in Sample 1) of one, two or three questionnaires (106 participants for the RRS-R, 116 participants for the PTQ and 113 participants for the Mini-CERTS). Participants (mean age = 27.11; *SD* = 8.93) were college students recruited through an Internet survey (*n* = 78) or students at the campus of the University of Gdansk, Poland (*n* = 88). The study was based solely on voluntary participation, consequently the recruitment through electronic and in-person methods were used jointly, with electronic methods used to supplement in-person data collection methods in order to increase the total number of participants. We did not find any systematic differences between data collected via electronic or in-person methods, and thus the data gathered from both samples were combined.

#### Sample 4

Participants (*n* = 206; 74 male and 132 female) filled in two of the Polish-language versions of questionnaires evaluating the RNT (57 participants for the PTQ and the RRS-R, 63 for the Mini-CERTS, and the PTQ, 86 for the Mini-CERTS and the RRS-R). Participants (mean age = 29.36; *SD* = 9.92) were college students recruited through an Internet survey, or students at the University of Gdansk campus. Similarly, as we did not find any systematic differences between data collected via electronic or in-in person methods, the data collected from both samples were combined.

### Measures

#### The ruminative response scale-revised (Treynor et al., 2003)

The RRS-R is a self-report questionnaire assessing depressive rumination. The scale is composed of 22 items. Participants are asked to evaluate on a 4 point Likert scale if they never, sometimes, often, or always, think or do each of the statements when they feel down, sad, or depressed. The scale enables calculation of a global depressive rumination score—a sum of the items. Additionally, the scale contains two subscales, each comprised of 5 items: brooding (Items: 5, 10, 13, 15, and 16), for example: “Think ‘What am I doing to deserve this?’ ” (Item 5) and reflection (Items: 7, 11, 12, 20, and 21), for example: “Go someplace alone to think about your feelings” (Item 21). The remaining 12 items are considered to be redundant with depressive symptomatology. The original version of the RRS-R presented satisfactory internal consistency (Cronbach's α = 0.77 for brooding and 0.72 for reflection). Concurrent validity of the RRS-R with depression is good: as expected, the Beck Depression Inventory (BDI) correlates with both, brooding (*r* = 0.44, *p* < 0.001) and reflection (*r* = 0.12, *p* < 0.001).

#### The perseverative thinking questionnaire (Ehring et al., 2011)

The PTQ is a self-report questionnaire assessing RNT from a content-independent perspective. In the questionnaire, participants are asked to describe how they typically think about negative experiences or problems, and rate the extent to which each statement applies to them when they think about negative experiences or problems, using the 5 point Likert scale from 0 (never) to 4 (almost always). The questionnaire is composed of a single second order factor: Repetitive Negative Thinking, and three lower-order factors. Lower-order factors include: core features of RNT (Items; 1, 2, 3, 6, 7, 8, 11, 12, and 13), for example, “The same thoughts keep going through my mind” (Item 1); unproductiveness of RNT (Items: 4, 9 and 14), for example: “I think about many problems without solving any of them” (Item 4); and capacity captured by RNT (Items: 5, 10, and 15), for example: “I can't do anything else while thinking about my problems” (Item 5). Internal consistency of the original version of the PTQ is very good (Cronbach's α = 0.94–0.95 for RNT; 0.92–0.94 for core features of RNT; 0.77–0.87 for unproductiveness of RNT; and 0.82–0.92 for mental capacity captured by RNT). The convergent validity of the PTQ with the RRS-R is good; total RNT score, core features of RNT, unproductiveness of RNT, and mental capacity captured by RNT are all correlated with brooding (RRS-R, correlation with each respectively, *r* = 0.63, 0.60, 0.61, 0.49, *p*s < 0.001), and reflection (*r* = 0.42, 0.42, 0.34, 0.35, respectively; *p*s < 0.001). The total RNT score and three sub dimensions are also correlated with depression symptomatology measured by the BDI (*r* = 0.54, 0.49, 0.54, 0.46, respectively; *p*s < 0.001), and with anxiety measured by the STAI-trait (*r* = 0.64, 0.60, 0.59, 0.50, respectively; *p*s < 0.001).

#### The mini-cambridge exeter repetitive thinking questionnaire (Douilliez et al., 2014)

The Mini-CERTS is a self-report questionnaire assessing processing mode in the RNT. The questionnaire assessed two dimensions: unconstructive repetitive thinking—abstract analytic thinking (AAT, items: 1, 3, 5, 6, 7, 10, 12, 14, and 15) for example: “My thinking tends to get stuck in a rut, involving only a few themes” (Item 1), and constructive repetitive thinking—concrete experiential thinking (CET; Items: 2, 4, 8, 9, 11, 13, and 16) for example: “I can grasp and respond to changes in the world around me without having to analyze the details” (Item 2). The original version of the Mini-CERTS has good internal consistency (Cronbach's α = 0.80 for AAT and 0.77 for CET) and good concurrent validity: AAT was correlated with the BDI, *r* = 0.44, *p* < 0.001 and the STAI-trait, *r* = 0.51, *p* < 0.001; CET is negatively correlated with the BDI, *r* = −0.18, *p* < 0.05. In the original study the convergent validity with the RRS-R was also good, AAT correlated with brooding *r* = 0.62, *p* < 0.001, but not with reflection *r* = 0.14, *ns.* CET was neither correlated with brooding or reflection (respectively: *r* = 0.01, *ns, r* = 0.10, *ns*).

### Procedure

The current study began with the evaluation of the Polish translation of each of the three scales. The translation was made by the first author and then consulted for clarity by three psychologists with a doctoral degree. In the evaluation phase the translation was evaluated by four independent judges from Sample 1 (both English and Polish-speaking psychology students from the SWPS University of Social Sciences and Humanities, Sopot). The judges evaluated the accuracy of the translation and the linguistic quality of the Polish items for each item on a dichotomous scale (good/need improvement). They also had the option to add their own alternative proposed translation if needed.

Next, two native English speakers evaluated the extent to which the back translation reflected the meaning of the original item. Ratings were made on a 0–2 scale, 0 if the translation did not fit the original version, 1 if it fit only partially, or 2 if it fit completely. They were instructed to refer only to the meaning in their evaluation. An option to report remarks or corrections concerning other lexical or grammatical aspects was given in the comments section.

Participants from Sample 3 then completed Polish-language and English-language versions of one of three scales to evaluate correlations between the versions and to assess basic psychometric proprieties of each scale. Finally, participants from the Sample 4 were asked to fill in two of the Polish-language versions of the scales to assess their convergent validity (participants were asked to fill in only two and not all three of the scales to reduce the drop-off due to the length of questionnaire^2^). The order of the questionnaires was randomized.

This study was carried out with written informed consent from all participants. Participants filling in an online version had to choose between two following possibilities: “Yes, I do agree to take part in the study described above” or “No, I do not want to take part in this study.” This study was conducted with the approval of the SWPS University of Social Sciences and Humanities Ethics Review Board, in accordance with the recommendations of the American Psychological Association and the Declaration of Helsinki.

## Results

Before conducting statistical analyses, the data were checked for missing items in order to exclude participants with more than two items missing for each scale; however, there were no cases in which more than two items were missing. In cases for which fewer than two items were missing, the missing items were replaced by the mean of the series. Interclass correlation (*ICC*; two-way mixed model) and structural equation modeling was used to compute relationships using a maximum likelihood (ML) estimation (see Table 1). Using a multitrait-multimethod approach (MTMM; Tomas et al., 2000), in addition to evaluating construct correlational relationships, repeated measures were also used. Sample 3 participants completed both the Polish and the English versions of the questionnaires; accordingly, in the analyses using Sample 3 data, we controlled for correlated errors of repeated measures.

**Table 1:** **Descriptive data for Polish-language and English-language versions: Correlation between Polish-language and English-language version of each dimension (ML estimation; MTMM approach)**.

**Variable (Questionnaire)**	**Polish version**			**English version**			**Correlation between Polish and English version**
	***M* (*SD*)**	**Cronbach's alpha**	***n***	***M* (*SD*)**	**Cronbach's alpha**	***n***	
Depressive rumination (RRS-R)	42.90 (11.05)	0.91	99	42.15 (12.91)	0.94	67	0.98^*^
Brooding (RRS-R)	9.79 (3.01)	0.74	99	9.50 (3.12)	0.79	67	0.98^*^
Reflection (RRS-R)	9.50 (3.14)	0.77	99	9.65 (3.35)	0.82	67	0.86^*^
RNT (PTQ)	27.17 (9.98)	0.92	111	25.72 (11.05)	0.94	66	0.97^*^
Core features of RNT (PTQ)	17.66 (6.39)	0.90	111	16.36 (6.72)	0.92	66	0.89^*^
Unproductiveness of RNT (PTQ)	4.73 (2.24)	0.64	111	4.82 (2.64)	0.81	66	0.89^*^
Mental capacity captured by RNT (PTQ)	4.56 (2.44)	0.76	111	4.63 (3.01)	0.79	66	0.93^*^
Abstract analytic thinking (Mini-CERTS)	21.76 (3.97)	0.69	110	21.50 (4.35)	0.74	72	0.95^*^
Concrete experiential thinking (Mini-CERTS)	18.79 (3.10)	0.61	110	18.52 (3.43)	0.70	72	0.93^*^

*RRS-R, The Ruminative Response Scale-Revised; RNT, Repetitive Negative Thinking; PTQ, The Perseverative Thinking Questionnaire; Mini-CERTS, The Mini-Cambridge Exeter Repetitive Thinking Scale*.

* *p < 0.001*.

### Translation accuracy

The accuracy of the translation and the linguistic quality of each item was evaluated by four independent judges. In regards to translation accuracy, zero items were evaluated as needing improvement by more than 25% of judges for the RRS-R (5 items for the PTQ and 5 for the Mini-CERTS). The linguistic quality of 1 item was assessed as needing improvement by more than 25% of judges for the RRS-R (4 for the PTQ and 4 for the Mini-CERTS). Those items that were evaluated as needing improvement were changed following independent judges' suggestions, and by the unanimous agreement of the two first authors. The back-translation assessed by two native English speakers (one specialist in psychology and one language specialist) revealed acceptable results (*ICC* = 0.76; *p* < 0.01) and there were no substantial recommendations for changing the meaning of the items.

### Consistency of responses between polish-language and english-language versions

In order to assess the fidelity of translation, and the understanding of the concepts conveyed by items in both language versions, the correlations between scores for each dimension for Polish-language and English-language were evaluated. We assessed these relationships in two ways. First, we computed *ICC*s in order to check how strongly responses to each pair of items in each questionnaire were correlated. One pair included a Polish-language item and a corresponding English-language version. Second, we computed a measurement model (ML) with correlated errors (MTMM approach) accounted for separately for each subscale of each questionnaire. We assumed that the second method was the best approach to control for errors that occurred when responding to the same item presented in two different language versions.

Each questionnaire demonstrated strong and significant *ICC*s. The RRS-R: the lowest *ICC* = 0.62 (*p* < 0.001), the highest was ICC = 1.00 (*p* < 0.001). The PTQ: the lowest *ICC* was 0.57 (*p* < 0.001), the highest *ICC* was 0.74 (*p* < 0.001), and the Mini-CERTS: the lowest *ICC* = 0.57 (*p* < 0.001), the highest *ICC* = 0.94 (*p* < 0.001).

The results of the ML analyses revealed that correlations for all of the dimensions were very strong (*r* = 0.86–0.98, *p*s < 0.001, see Table 1). Measurement model fit parameters for each subscale are presented in Table 2. Models were evaluated using the chi-square test statistic, the Comparative Fit Index (CFI), the Tucker-Lewis Index (TLI), the Root Mean Square Error of Approximation (RMSEA). Values larger than.90 of CFI and TLI, and.08 or lower of RMSEA indicate good model fit (Hu and Bentler, 1999).

**Table 2:** **Measurement model fit parameters for each dimension (ML estimation; MTMM approach) when testing consistency of responses between Polish-language and English-language versions**.

**Dimension**	**χ^2^(*df*)**	**χ^2^/*df***	**CFI**	**TLI**	**RMSEA (90% C.I.)**
Brooding	54.99^*^(29)	1.90	0.93	0.88	0.17 (0.10–0.24)
Reflection	36.04(29)	1.24	0.98	0.96	0.09 (0.01–0.19)
Core features of RNT	226.76^**^(125)	1.81	0.91	0.89	0.11 (0.09–0.13)
Unproductiveness of RNT	6.66(5)	1.33	0.99	0.98	0.07 (0.01–0.19)
Mental capacity captured by RNT	1.40(5)	0.28	1.00	1.00	0.01 (0.01–0.06)
Abstract analytic thinking	175.99^*^(125)	1.41	0.90	0.87	0.11 (0.07–0.14)
Concrete experiential thinking	126.58^**^(69)	1.83	0.82	0.77	0.15 (0.10–0.19)

*CFI, the Comparative Fit Index; TLI, the Tucker-Lewis Index; RMSEA, the Root Mean Square Error of Approximation*.

* *p < 0.01*,

** *p < 0.001*.

### Reliability (internal consistency)

The internal consistency was evaluated by computing Cronbach's alpha for each dimension. The Polish-language version of the RRS-R demonstrated good internal consistency, Cronbach's α = 0.77–0.91. Similarly, the internal consistency of the PTQ was also low but adequate, especially taking into account the low number of items in two of the PTQ dimensions: α = 0.64–0.92. The value of Cronbach's α for the Mini-CERTS dimensions acceptable though low, exceeding (Nunnally, 1967) > 0.60 criterion, α = 0.61 for CET and 0.69 for AAT, respectively.

In order to detect items affecting Cronbach's alphas, item-total correlation and Cronbach's alphas after suppression were computed (see Table 3). For the PTQ, all 15 items presented strong correlations and significant regression weights (*p*s < 0.001) between each item and the total RNT score and the three the PTQ dimensions. Suppression of these items did not improve the internal consistency measured by Cronbach's alpha. Similarly, each of the items related to RNT showed a significant correlation with the total score and with the respective dimensions as expected, and none of the item suppressions tested increased the Cronbach's alpha value. Correlation coefficients for the Mini-CERTS items 5 and 9 were not significant. Although suppressing item 5 does not increase Cronbach's alpha value for abstract analytic thinking dimension, suppressing item 9 seems to improve the value: 0.64 after suppressing the item compared to 0.61 for the dimension containing all 7 items. The low levels of reliability suggest a need for possible scale revision and reevaluation; however despite this, the standard items of the Mini-CERTS were retained for the remaining analysis to make the results more widely applicable by using the standard items.

Table 3**Item-total standardized regression weights (ML estimation, MTMM approach), item-total Pearson's correlation and Cronbach's alpha values after suppressing items**.

**Table:** 

**Item**	**Rumination (regression weight/Pearson correlation)**		**Brooding (regression weight/Pearson correlation)**		**Reflection (regression weight/Pearson correlation)**		**Total Cronbach's alpha after suppression of the item**	**Dimension Cronbach's alpha after suppression of the item**	***n***
RRS-R1	0.44^**^	0.40^b^					0.91		106
RRS-R2	0.44^**^	0.53					0.90		106
RRS-R3	0.58^**^	0.50					0.90		106
RRS-R4	0.54^**^	0.55					0.90		106
RRS-R5	0.49^**^	0.46	0.62^**^	0.48			0.91	0.71	106
RRS-R6	0.62^**^	0.63					0.90		106
RRS-R7	0.55^**^	0.53			0.67^**^	0.61	0.90	0.69	106
RRS-R8	0.57^**^	0.54					0.90		106
RRS-R9	0.54^**^	0.54					0.90		106
RRS-R10	0.51^**^	0.50	0.43^**^	0.39			0.90	0.74	106
RRS-R11	0.63^**^	0.60			0.73^**^	0.57	0.90	0.70	106
RRS-R12	0.22^*^	0.21			0.35^**^	0.34	0.91	0.78	106
RRS-R13	0.55^**^	0.53	0.59^**^	0.51			0.90	0.70	106
RRS-R14	0.64^**^	0.61					0.93		106
RRS-R15	0.56^**^	0.53	0.77^**^	0.61			0.90	0.66	106
RRS-R16	0.56^**^	0.55	0.64^**^	0.58			0.90	0.68	106
RRS-R17	0.74^**^	0.70					0.90		106
RRS-R18	0.70^**^	0.65					0.90		106
RRS-R19	0.49^**^	0.46					0.91		106
RRS-R20	0.51^**^	0.51			0.56^***^	0.52	0.90	0.72	106
RRS-R21	0.60^**^	0.57			0.78^**^	0.61	0.90	0.69	106
RRS-R22	0.66^**^	0.62					0.90		106

**Table:** 

**Item**	**RNT (regression weight/Pearson correlation)**		**Core features of RNT (regression weight/Pearson correlation)**		**Unproductiveness of RNT (regression weight/Pearson correlation)**		**Mental capacity captured by RNT (regression weight/Pearson correlation)**		**Total Cronbach's alpha after suppression of the item^a^**	**Dimension Cronbach's alpha after suppression of the item**	***n***
PTQ1	0.75^**^	0.69	0.81^**^	0.72					0.92	0.88	116
PTQ2	0.70^**^	0.67	0.77^**^	0.72					0.92	0.88	116
PTQ3	0.77^**^	0.72	0.79^**^	0.75					0.91	0.88	116
PTQ4	0.51^**^	0.50			0.51^**^	0.41			0.92	0.61	116
PTQ5	0.66^**^	0.64					0.71^**^	0.59	0.92	0.69	116
PTQ6	0.66^**^	0.64	0.75^**^	0.70					0.92	0.87	116
PTQ7	0.60^**^	0.61	0.64^**^	0.63					0.92	0.89	116
PTQ8	0.78^**^	0.73	0.67^**^	0.63					0.91	0.89	116
PTQ9	0.55^**^	0.58			0.81^**^	0.53			0.92	0.45	116
PTQ10	0.69^**^	0.67					0.72^**^	0.59	0.92	0.68	116
PTQ11	0.73^**^	0.67	0.70^**^	0.67					0.92	0.89	116
PTQ12	0.52^**^	0.53	0.60^**^	0.57					0.92	0.90	116
PTQ13	0.70^**^	0.70	0.62^**^	0.62					0.92	0.89	116
PTQ14	0.59^**^	0.58			0.57^**^	0.43			0.92	0.48	116
PTQ15	0.68^**^	0.67					0.74^**^	0.60	0.92	0.67	116

**Table:** 

**Item**	**Abstract analytic thinking (regression weight/Pearson correlation)**		**Concrete experiential thinking (regression weight/Pearson correlation)**		**Dimension Cronbach's alpha after suppression of the item**	***n***
Mini-CERTS3	0.66^**^	0.49			0.61	111
Mini-CERTS4			0.72^**^	0.52	0.50	111
Mini-CERTS5	0.17^*^	0.25			0.69	111
Mini-CERTS6	0.47^**^	0.42			0.63	111
Mini-CERTS7	0.38^*^	0.33			0.65	111
Mini-CERTS8			0.73^**^	0.50	0.52	111
Mini-CERTS9			0.05	0.10	0.64	111
Mini-CERTS10	0.45^**^	0.36			0.64	111
Mini-CERTS11			0.36^*^	0.27	0.56	111
Mini-CERTS12	0.45^**^	0.38			0.64	111
Mini-CERTS13			0.31^*^	0.25	0.60	111
Mini-CERTS14	0.26^*^	0.23			0.67	111
Mini-CERTS15	0.48^**^	0.32			0.65	111
Mini-CERTS16			0.51^**^	0.37	0.56	111

a Computed only for scales admitting total score as an assessment of RNT, namely the PTQ and the RRS-R

b *p-values are the same for regression weights and rs*.

*RRS-R, The Ruminative Response Scale-Revised; RNT, Repetitive Negative Thinking; PTQ, The Perseverative Thinking Questionnaire; Mini-CERTS, The Mini-Cambridge Exeter Repetitive Thinking Scale*.

* *p < 0.05*.

** *p < 0.01*.

*** *p < 0.001*.

### Factorial validity

We performed exploratory factor analyses (EFA) to test if the structure of each Polish-language version of a questionnaire was similar to the English-language version. Principal axis model was used to extract factors with oblimin rotation (delta = 0). Each model was restricted to the number of factors representing the original versions of each questionnaire.

Initial results of the RRS-R results showed good sampling adequacy (*KMO* = 0.83). Two factors were extracted. The first factor explained approximately 41% of variance, whereas the second factor approximately 9% (eigenvalues were higher than 1). The first factor represented the brooding dimension and covered 4 of 5 items compared with the original version (5, 13, 15, and 16). The second factor represented the reflection dimension and covered 5 of 5 item compared with the original version.

Results of the PTQ results showed good sampling adequacy (*KMO* = 0.90). Three factors were extracted. The first factor explained approximately 45% of variance, the second factor approximately 8% (eigenvalues were higher than 1), and the third 4% (eigenvalue lower than 1). The first factor represented the Unproductiveness dimension and covered 3 of 3 items compared with the original version. The second factor represented the core features of RNT dimension and covered 6 of 9 original items (1, 2, 3, 6, 7, and 12). The third factor represented mental capacity captured by RNT subscale and covered 2 of 3 original items (5 and 15).

The EFA of the Mini-CERTS revealed low sampling adequacy (*KMO* = 0.67). Two factors were extracted. The first factor explained approximately 18% of variance, whereas the second factor approximately 15% (eigenvalues were higher than 2). The first factor represented the abstract analytic thinking subscale and covered 7 of 9 items compared with the original version (1, 3, 6, 7, 10, 12, and 15), whereas the second factor represented the concrete experiential thinking dimension and covered 5 of 7 original items (4, 8, 11, 13, and 16).

### Convergent validity—comparison between the three scales' factor structures

The convergent validity of the scales was assessed by computing the correlations between dimensions of the RRS-R, the PTQ, and the Mini-CERTS (see Table 4). Confirming the theoretical prediction, the brooding score was positively correlated to RNT, all the sub dimensions of the PTQ, and AAT from the Mini-CERTS. Finally, also in accordance with the theoretical predictions, AAT was negatively correlated with CET.

**Table 4:** **Correlation between variables in the Polish-language version of questionnaires (maximum likelihood method estimation)**.

**Variable (Questionnaire)**	**1**	**2**	**3**	**4**	**5**	**6**	**7**	**8**
Depressive rumination (RRS-R)	–							
Brooding (RRS-R)	0.67^***^	–						
Reflection (RRS-R)	0.98^***^	0.56^**^	–					
RNT (PTQ)	0.73^*^	0.66^**^	0.67^**^	–				
Core features of RNT (PTQ)	0.69^*^	0.62^**^	0.72^***^	0.95^***^	–			
Unproductiveness of RNT (PTQ)	0.80^*^	0.77^**^	0.52^*^	0.97^***^	0.77^***^	–		
Mental capacity captured by RNT (PTQ)	0.71^*^	0.61^**^	0.62^*^	0.86^***^	0.86^***^	0.96^***^	–	
Abstract analytic thinking (Mini-CERTS)	0.74^**^	0.91^***^	0.48^*^	0.80^**^	0.74^***^	0.85^**^	0.79^**^	–
Concrete experiential thinking (Mini-CERTS)	–0.41^*^	–0.42^*^	–0.22	–0.05	0.06	–0.32	–0.26	–0.43^*^

*RRS-R, The Ruminative Response Scale-Revised; RNT, Repetitive Negative Thinking; PTQ, The Perseverative Thinking Questionnaire; Mini-CERTS, The Mini-Cambridge Exeter Repetitive Thinking Scale*.

* *p < 0.05*,

** *p < 0.01*,

*** *p < 0.001*.

## Discussion

The aim of the present study was to translate and conduct a validation of three different questionnaires assessing RNT: the RRS-R, the PTQ and the Mini-CERTS and to compare the main characteristics of those scales. The three questionnaires presented above evaluate the same process (RNT), yet each provides different options due to the specificity of each scale, making it possible to adjust the choice of scale for a precise clinical or research purpose. This is particularly crucial given the lack of available instruments for international multilingual comparisons, particularly for clinical research uses in which there is a gap between increasing interest in this area and lack of instrumentation.

The correlations between RRS-R, PTQ, and Mini-CERTS support the theoretical hypothesis. As expected, the dimensions of brooding, RNT, and AAT, that can be classified as unconstructive repetitive thinking (Nolen-Hoeksema, 1991; Watkins, 2008; Ehring et al., 2011), were correlated. Although those three dimensions refer to different characteristics of RNT (depressive rumination, transdiagnostic RNT, or processing mode), those characteristics overlap and there is a clear impact of this redundancy in the results. It is particularly noteworthy that the three questionnaires are not interchangeable (the RRS-R, the PTQ, and the Mini-CERTS), thus the availability of three scales gives a choice to researchers and clinicians. The development of a transdiagnostic assessment of RNT seems to be of particular interest for research expansion across clinical in order to better understand the mechanisms that underlie many pathologies. Although the link between mood disorders and RNT is widely explored in correlational studies, the research on the impact of RNT on other psychological disorders and the number of experimental studies on RNT is still expanding.

The RRS-R, the most widely used evaluation of RNT, seems to be ideal for evaluating RNT in depressed patients. Initial results evaluating the Polish-language version of the RRS-R are promising, both the internal consistency and convergent validity appear to be good. The PTQ evaluates basic characteristics of RNT in a content and disorder independent perspective. The evaluation of RNT, of both rumination and worries as one process, seems to be especially interesting in the transdiagnostic use for comorbid disorders. The psychometric qualities of the Polish-language version of the PTQ are good. Since its publication in 2011, the PTQ already has four translated versions (English, German, Dutch, and Portuguese), which suggests that there may be an interest in both the scientific and clinical communities for this scale (Ehring et al., 2011, 2012; Macedo et al., 2015). Polish versions of the RRS-R, PTQ, and Mini-CERTS have a similar psychometric parameters (internal consistency, factorial and diagnostic validity) to other language versions. Tests of consistency of responses between Polish-language and English-language versions conducted in a MTMM approach with ML estimation showed rather average fit of the models to the data. The CFI values for each dimension, excluding the concrete experiential thinking dimension, were above 0.90 threshold (cf. Hu and Bentler, 1999). The fit of the models was driven by a relatively low statistical power. Future studies should test the models in larger samples.

To our knowledge the present study is the first to compare Mini-CERTS to another measure of transdiagnostic RNT. The results are promising, in accordance with theoretical predictions of processing mode theory, AA seems to be positively correlated to the total score and all dimensions of the PTQ, while CE—an adaptive processing mode—is not related to RNT as measured by the PTQ. Additionally, the negative correlation between AA and CE corroborates the prediction of processing mode theory and interacting cognitive subsystems model (Barnard and Teasdale, 1991), confirming that the two processing modes are mutually exclusive. This postulate is also a cornerstone of concreteness training (Watkins et al., 2011).

### Implications and future directions

The studies inducing RNT are crucial for the development and improvement of RNT-focused clinical interventions. In addition, in experimental studies it is necessary to evaluate the interaction between experimental induction and trait RNT. Consequently the choice of an adapted assessment of trait RNT seems to be key.

Of particular interest for research and rumination focused CBT is the use of the Mini-CERTS, the transdiagnostic evaluation of information processing in RNT. The method has a definitive advantage of assessing both types of RNT processing mode. The Mini-CERTS uniquely assesses constructive and unconstructive RNT in terms of abstract and concrete processing modes. This assessment seems to be particularly relevant for clinical and translational researchers, as therapeutic intervention for maladaptive RNT (e.g., concreteness training, Watkins et al., 2011) is based on the distinctive functionality of two alternative processing modes. The scale also seems to have an excellent concurrent validity (in the original version and published research; Di Schiena et al., 2012; Kornacka and Douilliez, 2014). The results of the present study, however, suggest that it might be necessary to adjust the structure of the questionnaire by adding more items in order to improve the internal consistency. In the research published so far the internal consistency of Mini-CERTS is variable, with the Cronbach's α from 0.60 to 0.89 (Di Schiena et al., 2012, 2013; Douilliez and Philippot, 2012; Kornacka and Douilliez, 2014; Pitance, 2014).

It is noteworthy that the suppression of item 9 from the concrete experiential thinking improved the internal consistency of the dimension measured by Cronbach's α. However, item 9 is the only item in the subscale referring directly to the mindful attentional focalization on the present moment “I seem to be engaged in and directly in touch with what is going on around me.” The other items on this dimension refer more to the ease of processing adjustment to the ongoing situation. The content of the item represents one of the core feature of adaptive repetitive thinking according to processing mode theory (Watkins, 2011). Thus, suppression of such an essential concept should be avoided.

An important factor worth taking into account while considering possible reasons for low internal consistency of Mini-CERTS is participant metacognitive skill. Although the scales like RRS and PTQ directly assess the level of maladaptive repetitive thinking, the Mini-CERTS goes beyond and aims at evaluating the processing mode. The importance of metacognition was explored in the perspective of positive vs. negative beliefs on repetitive negative thinking (Papageorgiou and Wells, 2003), though, it is important to note that metacognitive skills might also be involved in the assessment, particularly in the processing mode context. It seems much easier to assess whether or not (and to what extent) one uses repetitive negative thinking than to assess whether that repetitive thinking involves abstract or concrete processing. Evidence corroborating this hypothesis includes the crucial role of psychoeducation within concreteness training, for the purpose of teaching patients to detect different types of repetitive negative thinking (Watkins et al., 2009, 2011). Difficulties with this metacognitive self-evaluation might be particularly relevant for the linkage of concrete experiential processing to the lower level, automatic processes making them consequently less available to verbalization (Vallacher and Wegner, 1989; Watkins, 2011). Considering the utility of the Mini-CERTS, further studies should explore how participants/patients metacognitive skills affect the use of the Mini-CERTS and how taking those skills into account during the evaluation might improve the internal consistency of the method.

The use of the Mini-CERTS may be of particular interest to clinical researchers interested in studying concreteness training because it is an intervention aiming at enhancing the use of concrete processing instead of automatic use of abstract processing. By design, those two processing modes correspond exactly to the two Mini-CERTS' dimensions. Thus, Mini-CERTS seems to be appropriate as a measure of therapeutic progress on these distinct dimensions, which no other scale can currently assess. First, it enables one to measure directly whether the one of the key goals of concreteness training is achieved (i.e., enhancing the use of concrete experiential thinking). Second, it enables measurement of the reduction in the automatic use of abstract evaluative repetitive thinking as a maladaptive emotional regulation strategy. Finally, researchers might also further investigate the hypothesized mechanisms behind processing mode theory suggesting that the two processing modes are interdependent. Among available RNT measures, the Mini-CERTS seems to be the only one to include the requisite specificity that assesses not only the level of the RNT use but also the processes underling RNT, and which could be applied at the baseline as well as after RNT-focused interventions are complete.

Furthermore, initial results in clinical settings have already shown the predictive validity of the questionnaire, measured so far for depressive/dysphoric symptomatology (Di Schiena et al., 2012, 2013; Kornacka and Douilliez, 2014). Abstract analytic thinking assessed by the Mini-CERTS seems to systematically predict the level of depressive symptomatology, contrary to the concrete experiential thinking that is either not (or is negatively) correlated to this psychological disorder. Considering the transdiagnostic feature of the assessment provided by the Mini-CERTS, it seems of wider interest to further develop evidence of predictive validity for other psychological disorders.

As suggested by the possible inadequacies of the Mini-CERTS scale evident in our data and elsewhere (Di Schiena et al., 2012; Kornacka and Douilliez, 2014), the factor structure of the Mini-CERTS needs to be reexamined in future studies to potentially revise and improve the scale. Particularly because of the novelty of the questionnaire—it has been used only in a few published studies, most of them based on non-clinical samples, and all using the French version of the scale (cf. Di Schiena et al., 2012, 2013; Kornacka and Douilliez, 2014). More multi-language studies are necessary to fully examine the psychometric parameters of the questionnaire.

The results of the current study also provide information on the psychometric parameters of each questionnaire (RRS-R, PTQ, Mini-CERTS). These data could be useful for meta-analyses and for evaluating reproducibility. Given the rising importance of reproducibility of research findings in the wider psychological literature, and recent concerns raised regarding the value of previous findings, the results at hand are important to strengthen replication efforts in the international community focused on measurement in psychopathology.

## Conclusion

The present paper provides a comparison of the structure, an evaluation of translation accuracy, and validation of three RNT scales adapted for Polish language use. Future studies should continue multi-language validation of the RRS-R, the PTQ and the Mini-CERTS, with a confirmatory factor analysis to determine if there are any structural differences due to linguistic patterns to compare across languages (cf. Roger et al., 2001); this is particularly important for the newly developed scales like the PTQ or the Mini-CERTS, along with a concurrent validity assessment in both the healthy and the clinical populations. Taking into account the limited availability of transdiagnostic RNT measures in clinical psychology and the lack of systematic comparison between the measures, the current research can address the need of providing this first evaluation of the scale properties to boost RNT research not only in the Polish population, but also worldwide.

## Author contributions

MK: conception and design; acquisition, analysis, and interpretation of results; writing manuscript. JB: conception and design; analysis and interpretation of results; writing manuscript. RL: interpretation of results; revising and writing manuscript.

## Funding

This research was supported by the Young Researchers International Mobility Grant (CM/YB 3273) attributed to MK by the Council of Nord-Pas-de-Calais Region, France.

### Conflict of interest statement

The authors declare that the research was conducted in the absence of any commercial or financial relationships that could be construed as a potential conflict of interest.

## Footnotes

^1^In countries actively conducting rumination experimental research, like English, French, German, or Dutch-speaking countries one can count up to 6 questionnaires assessing repetitive negative thinking. Comparatively, in Polish there is one scale assessing rumination in social context (Baryla and Wojciszke, 2005) and the only clinical scale assessing rumination is a translation of Rumination-Reflection Questionnaire (RRQ; Trapnell and Campbell, 1999; Radon, 2014 for the Polish validation), although, the RRQ is quite rarely used in recent rumination research. A Google Scholar search revealed and the article validating RRQ was cited only 421 times in comparison with the revised version of RRS-R which was cited 1370 times in the same period (between 2010 and 2015, as of January 29, 2015; not counting the citations of the original, non-revised version of RRS).

^2^Pilot studies indicated that dropout rates were particularly high due to use of a solely volunteer sample. Thus, to decrease the amount of incomplete questionnaires yet still maintain the ability to compare results across questionnaires. Participants were only asked to complete two questionnaires. Furthermore, to maintain an adequate sample size, both internet-based and in-person data collection methods were used.
